# Eight years of the East African Community Medicines Regulatory Harmonization initiative: Implementation, progress, and lessons learned

**DOI:** 10.1371/journal.pmed.1003134

**Published:** 2020-08-12

**Authors:** Jane H. Mashingia, Vincent Ahonkhai, Noel Aineplan, Aggrey Ambali, Apollo Angole, Mawien Arik, Samvel Azatyan, Peter Baak, Emmanuel Bamenyekanye, Aimable Bizoza, Chimwemwe Chamdimba, Petra Doerr, Adam Fimbo, Alex Gisagara, Hidaya Hamad, Rachelle Harris, Dan Hartman, Joseph Kabatende, Charles Karangwa, Agnes Sitta Kijo, Murray Lumpkin, Shani Maboko, David Matle, Apollo Muhairwe, John Patrick Mwesigye, Bonaventure Nyabenda, Alexander Schulze, Andreas Seiter, Gordon Sematiko, Margareth Sigonda, Hiiti Sillo, Burhani Simai, Fred Siyoi, Stanley Sonoiya, Paul Tanui, Mike Ward, Felistas Yano, David Mukanga

**Affiliations:** 1 East African Community Secretariat, Arusha, Tanzania; 2 Gwynedd Consultancy, LLC, Philadelphia, Pennsylvania, United States of America; 3 National Drug Authority, Kampala, Uganda; 4 African Union Development Agency–New Partnership for Africa’s Development, Midrand, South Africa; 5 Drug and Food Control Authority, Juba, South Sudan; 6 World Health Organization, Geneva, Switzerland; 7 Directorate of Pharmacy, Medicines, and Laboratories, Bujumbura, Burundi; 8 Rwanda Food & Drugs Authority, Kigali, Rwanda; 9 Petra Doerr Consulting Ltd., Herznach, Switzerland; 10 Tanzania Medicines and Medical Devices Authority, Dar Es Salaam, Tanzania; 11 Zanzibar Food & Drug Agency, Zanzibar City, Zanzibar; 12 Harris Access Consulting Ltd, London, United Kingdom; 13 Bill & Melinda Gates Foundation, Seattle, Washington, United States of America; 14 World Bank, Washington DC, United States of America; 15 Swiss Agency for Development and Cooperation, Bern, Switzerland; 16 Pharmacy & Poisons Board, Nairobi, Kenya

## Abstract

Jane H. Mashingia and colleagues reveal the progress made to date for the East African Community Medicines Regulatory Harmonization initiative.

**Summary points**

- Access to essential medicines is a key pillar of any health system seeking to deliver universal health coverage. Science-based, independent regulation of medical products is a critical part of ensuring that only quality essential medicines reach the patients who need them.
- In this article, we explore the progress the East African Community’s Medicines Regulatory Harmonization (EAC MRH) initiative, launched in 2012, has made toward its goal of improving access to essential medicines. The initiative’s initial focus was on registering generic medicines, with a plan to expand to other classes of medical products, as well as to other regulatory functions.
- From 2012 to 2017, the timeline for national assessments of medicinal product applications decreased from roughly 24 months to 8–14 months, if products were assessed through the new joint assessment process (involving 2 or more national medicines regulatory authorities).
- Since 2015, the initiative has conducted 10 joint product assessment sessions in which 83 medicinal product applications were considered, resulting in the recommendation of 36 products for registration by EAC Partner States.
- Overall, the median timeline for a joint assessment, from submission of the application through final decision, has been a little over a year (372 days); 170 of these days represent time used by manufacturers to answer queries. However, the median timeline for a joint assessment in 2019 was only 240 days, indicating that the process has become more efficient.
- Shifting from relying on donor support to becoming self-sustaining remains a challenge for the EAC MRH initiative.

## Introduction

Access to essential medicines is a key pillar of any health system seeking to deliver universal health coverage. Science-based, independent regulation of medical products is a critical part of ensuring that only quality essential medicines reach the patients who need them. However, such regulation needs to be conducted in a transparent, efficient, accountable, and predictable manner to have a positive impact on public health. National medicines regulatory authorities (NMRAs) have their mandate codified in national legislations and put into practice by applying a set of internationally recommended core regulatory functions. According to the World Health Organization (WHO) [1], NMRAs contribute to promoting and protecting public health and safety by ensuring that.

- medicines are of the required quality, safety, and efficacy;
- health professionals and patients have the necessary information to enable them to use medicines rationally;
- medicines are appropriately manufactured, stored, distributed, and dispensed;
- illegal manufacturing and trade are detected and adequately sanctioned;
- promotion and advertising are fair, balanced, and aimed at rational drug use; and
- access to medicines is not hindered by unjustified regulatory work.

In 2012, the East African Community (EAC)—a regional economic community consisting, at that time, of Burundi, Kenya, Rwanda, the United Republic of Tanzania, and Uganda—recognized that, though its NMRAs were fulfilling many of these important functions, much room for improvement still existed with regard to improving its citizens’ access to essential medicines. In particular, the EAC hoped to improve access to medicines by making the marketing authorization application process for manufacturers more efficient by increasing the speed at which it reviewed applications, without decreasing rigor, and by modernizing its processes and procedures. To work toward these goals, it initiated the EAC Medicines Regulatory Harmonization (MRH) initiative.

The initiative reached a major milestone in 2015, when Roche applied to market 2 oncology medicines, bevacizumab and trastuzumab, in the EAC. Neither medicine was new. Bevacizumab was first approved by the United States Food and Drug Administration in 2004 and trastuzumab in 1998 [2,3], and both medicines were on WHO’s list of essential medicines [4]. However, neither medicine had been registered in any EAC country. Before the EAC MRH initiative was established, a company would have had to complete different registration applications for each EAC country. Thanks to the initiative, however, Roche was able to apply for a single region-wide joint assessment of the medicines, the first ever performed in the EAC. The medicines were recommended for registration throughout the region, and using the positive joint assessment and recommendation, they were registered in mainland Tanzania within 4 months of application for joint assessment [5]. This represented a substantial improvement over the 2-year average registration time in the region before the initiative began. Afterward, the medicines were registered by Kenya and Uganda (although the medicines were eligible for registration in all EAC countries, the manufacturer decided to register them in only 3). Because of this new regional approach to product assessment, these medicines were available in EAC countries sooner than they would have been otherwise. This benefited the patients, who gained earlier access to the medicines; the manufacturer, which was able to more efficiently register its medicines in multiple countries (while it had patent protection on the medicines); and the NMRAs of the EAC countries, which saved time and resources by conducting a single joint assessment rather than multiple national assessments.

In this review article, we will further explore what the EAC MRH initiative, which launched in 2012, has accomplished in its 8 years of existence. In addition to the wealth of personal experience with the initiative that the authors of this article have, we draw on information from an evaluation completed in late 2017 by the Boston Consulting Group (BCG), which interviewed staff from EAC Partner States’ NMRAs and Ministries of Health; industry representatives from multinational companies and local medicines importers and manufacturers that had used the new processes; and staff from key stakeholders such as the EAC Secretariat, the African Union Development Agency–New Partnership for Africa’s Development (AUDA-NEPAD), the World Bank, donors, and technical partners such as WHO. By sharing the most successful and challenging aspects of the initiative, we hope to help other regions that are planning similar programs—and to encourage the community, especially individuals from low- and middle-income countries, to develop and share their own ideas regarding the harmonization, alignment, and optimization of regulatory processes and technical standards at a regional level.

## Progress toward the major goals of the EAC MRH initiative

As described in an accompanying article in this collection, *Coming together to improve access to medicines*: *The genesis of the East African Community’s Medicines Regulatory Harmonization initiative* [6], the overarching vision of the EAC’s MRH initiative was to increase the number of quality medicines registered in the region by (1) simplifying the application process for manufacturers and (2) increasing the efficiency with which applications were assessed by Partner States—without sacrificing product efficacy, safety, or quality. In pursuit of this vision, the initiative originally decided to focus on

- Developing and implementing
  - A Common Technical Document (CTD), based on the International Council for Harmonisation of Technical Requirements for Pharmaceuticals for Human Use (ICH) CTD, that manufacturers could use to register medicines within any EAC Partner State
  - A common information management system (IMS) for medicines registration that would link all Partner States, as well as the EAC Secretariat
  - A quality management system (QMS) in each NMRA, to ensure that each Partner State carried out regulatory activities in a uniform and rigorous manner
- Building the EAC’s regional and national capacity to implement registration processes and harmonize and align technical standards; and
- Developing and implementing a framework for Partner States to eventually recognize the regulatory findings and decisions of their neighbors.

Although its initial focus was on registering generic medicines, the plan was for the initiative to later expand to other classes of medical products, such as biosimilars, vaccines, and medical devices, as well as to other regulatory functions, such as clinical trials oversight and pharmacovigilance. Finally, the EAC MRH initiative was expected to identify a funding mechanism that would allow it to sustain and broaden its regulatory activities after the catalytic donor support available for the first 5 years expired.

### Successes on the way to MRH

The EAC MRH initiative succeeded in increasing the efficiency with which registration applications for medicines were assessed. From 2012 to 2017, the timeline for national assessments (those carried out by single NMRAs) decreased from roughly 24 months at baseline (estimated with the help of past applicants) to 8 to 14 months in Kenya and 10 to 12 months in Tanzania (range based on median and average timelines) [7]. In Uganda, the timeline decreased from an estimated 18 months at baseline to 14 to 16 months [7]. Other countries in the EAC did not assess product dossiers for registration prior to the EAC MRH initiative. Indeed, this is one of the achievements of the EAC MRH initiative: It has enabled these countries to set up their own fit-for-purpose marketing authorization systems.

These shorter timelines were possible, in part, because in 2015, the initiative adopted a modified version of the CTD developed by the ICH. Manufacturers could use this CTD to apply for registration of medicines in any EAC Partner State. The initiative’s Medicines Evaluation & Registration Working Group, led by Tanzania’s NMRA, created this CTD as part of the program’s larger mandate of harmonizing technical requirements, standards, and standard operating procedures (SOPs) for medicines assessment and registration across the region [8]. Because the EAC’s CTD is based on the formats used by ICH and the WHO’s Prequalification Programme, EAC Partner States can easily leverage dossiers previously submitted to other regulatory authorities, such as the WHO, US Food and Drug Administration, or European Medicines Agency. With the consent of the applicant, regulatory agencies can enter into confidentiality agreements with other regulatory agencies to share assessment reports on dossiers they have evaluated; however, if the formats used for dossiers are different from the CTD, it would be cumbersome to follow the reviews undertaken by agencies sharing their reports. In addition, manufacturers can easily file dossiers across multiple territories if they all use the same international standards and formats; not having to develop tailored applications saves the manufacturers significant resources. Between 2015 and 2017 alone, more than 3,500 applications were submitted to the NMRAs of EAC Partner States using the new CTD format. These applications were submitted directly to the various NMRAs outside the joint assessment process. In interviews conducted by BCG, multinational companies reported that the transition to the new CTD format was easy, as they were already accustomed to preparing dossiers in this format for other markets. Local companies found the transition to the new CTD format more challenging, as it was unfamiliar and often more comprehensive than the formats they had used previously. In general, though, both types of companies reported that the new CTD format was an improvement, as applicants no longer had to prepare different dossiers for each country in the EAC.

**Fig 1:**
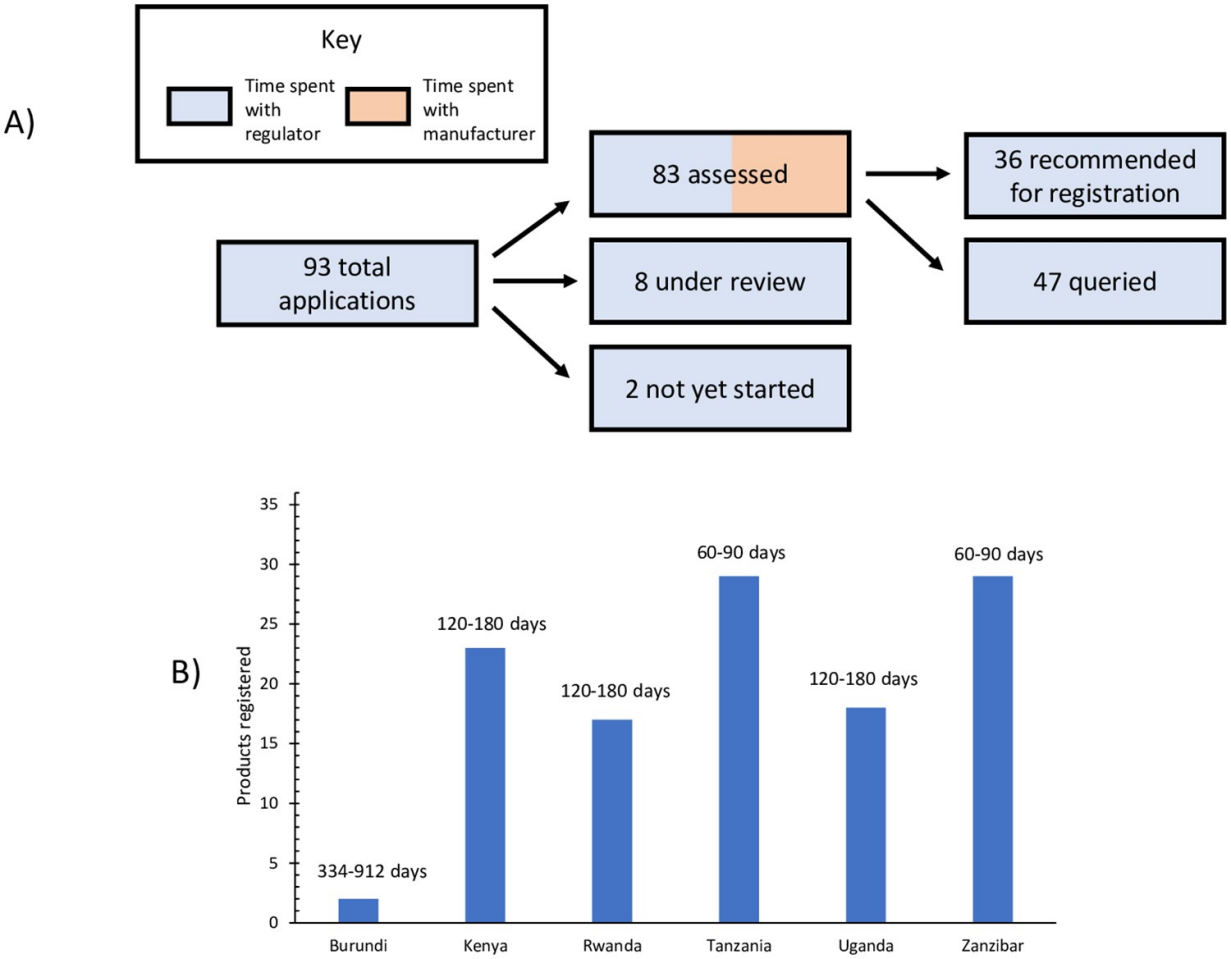
Outcomes for the joint assessment pathway of the East African Community’s Medicines Regulatory Harmonization initiative, from 2015, when the first applications were submitted, through August 2019. (A) Number of applications submitted, assessed, and recommended for registration. The relative amount of time spent with the regulator versus the manufacturer (while addressing queries from the regulator) is shown for assessments. (B) Number of medicines registered by each of the East African Community’s individual national medicines regulatory authorities, of the 36 recommended to date through the joint assessment pathway. The numbers above each Partner State’s name represent the range of times elapsing from joint assessment recommendation to product registration. These ranges include, where relevant, the time that a manufacturer took to submit an application and payment to the Partner State’s national medicines regulatory authority after receiving a joint assessment recommendation. Some smaller markets, such as Burundi, have received fewer registration applications from manufacturers following the joint assessment process. South Sudan, a new member of the initiative, has not yet started to register products and thus is not shown here.

**Fig 2:**
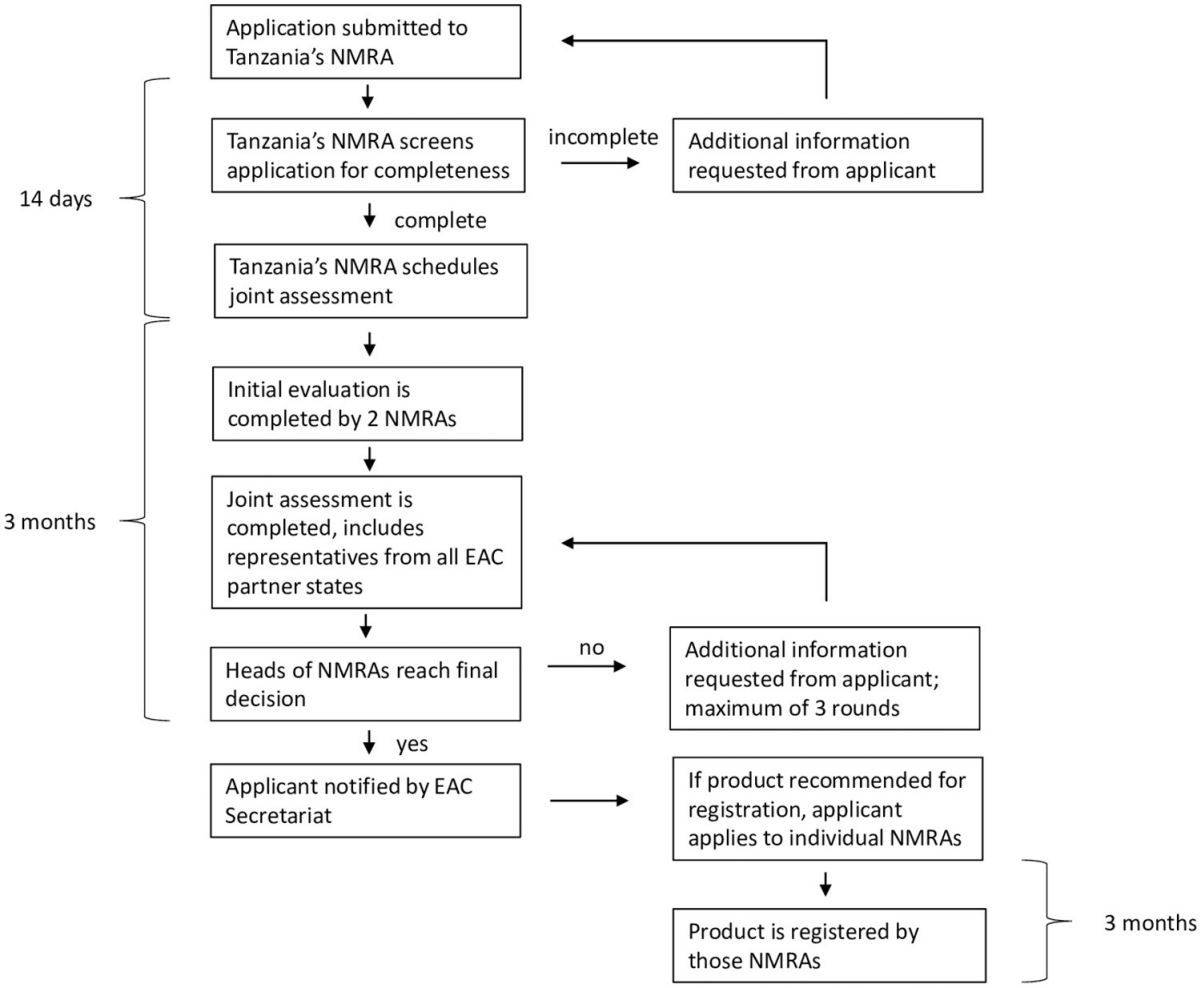
The joint product assessment pathway of the EAC’s Medicines Regulatory Harmonization initiative. EAC, East African Community; NMRA, national medicines regulatory authority.

The EAC MRH initiative also began conducting joint regulatory activities. Since 2015, the initiative has conducted 10 joint product assessment sessions, in which it has assessed 83 medicinal product applications, resulting in the recommendation of 36 products for registration by EAC Partner States (Fig 1). For each product subjected to joint assessment, 2 NMRAs are assigned to perform an initial assessment and share their findings, which are then discussed by all EAC NMRAs via joint assessment sessions held by the Medicines Evaluation & Registration Working Group (Fig 2). Overall, the median timeline for a joint assessment, from submission of the application through final assessment decision, has been a little over a year (372 days); 170 of these days represent the time used by manufacturers to answer queries. However, the median timeline for a joint assessment in 2019 was only 240 days, indicating that the process has become more efficient.

Once a product has been recommended for registration through the joint assessment process, registration by individual NMRAs should be swift, with a target timeline of 3 months or less. However, as in the example of bevacizumab and trastuzumab described previously, many manufacturers whose products have undergone joint assessment have decided not to register those products in all EAC Partner States (Fig 1). Reasons for this failure to register vary. An applicant may not be ready or desire to establish business in a given country for commercial reasons, for example. Or an applicant may not register its product in Zanzibar because once a product is registered in Tanzania, Zanzibar automatically recognizes the registration, so a second registration is unnecessary. To date, the maximum number of NMRAs that have registered a single product following joint assessment is 5, and this occurred for only 1 product (cetuximab). Furthermore, although Tanzania’s rapid approval of bevacizumab and trastuzumab following joint assessment demonstrates what can be accomplished with the new system, there has been a great deal of variation in the time it takes to finalize national registration. When manufacturers seek to register products with individual NMRAs after receiving a recommendation for registration, some have reported significant delays—sometimes of over a year [9]. The range of time to registration after a positive joint assessment is 60 to 90 days for the NMRAs of Tanzania and Zanzibar (meeting the 3-month target) and 120 to 180 days for the NMRAs of Kenya, Rwanda, and Uganda (exceeding the 3-month target). The range of time to registration for Burundi is 334 to 912 days, though these numbers reflect the registration of just 2 products. It should be noted that all of the time ranges given include the time that companies take to submit national applications and pay fees following a joint recommendation, as well as the time taken by regulators to process national applications.

**Fig 3:**
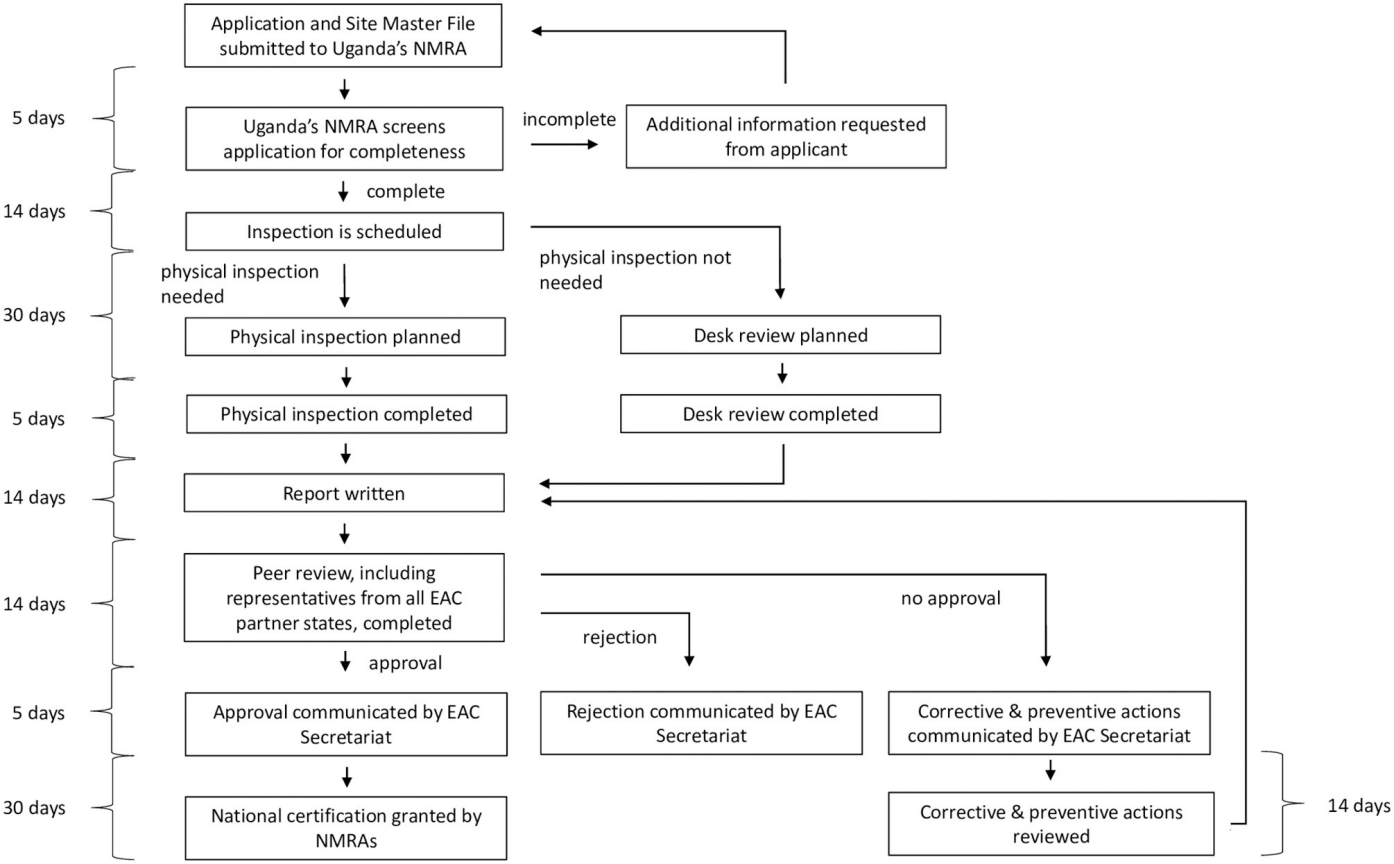
The joint good manufacturing process inspection pathway of the EAC’s Medicines Regulatory Harmonization initiative. EAC, East African Community; NMRA, national medicines regulatory authority.

Meanwhile, joint good manufacturing practice (GMP) inspections began in 2016. First, the initiative’s GMP Inspection Working Group, led by Uganda’s NMRA, established guidelines and SOPs for both national and joint inspections of factories in the region, based on WHO guidelines [10]. These SOPs were used during the 15 joint GMP inspections conducted thus far, resulting in 14 joint recommendations for approval. Similar to joint product assessments, joint GMP inspections are assigned to inspectors from 2 NMRAs, who share their findings with the EAC’s other NMRAs via the GMP Inspection Working Group (Fig 3). Most joint inspections have been performed on facilities that produce low-cost generic medicines. In 2019, for example, all 6 joint inspections performed to date have been on facilities that produce generics.

Joint assessments and inspections conferred benefits on both applicants and the EAC’s NMRAs. As described in the aforementioned examples, using the joint process, companies seeking to register a medicine in the EAC could undergo fewer assessments and inspections. Moreover, when the program worked as intended, registration in EAC Partner States proceeded smoothly, using the same applications and relying upon the information and recommendations from joint assessments or inspections. NMRAs, for their part, were able to work more efficiently, as the opportunity to rely on findings from joint assessments and inspections meant they need not perform their own assessments or inspections for every application received. In addition, joint activities facilitated capacity building and trust building between the NMRAs acting as assessors or inspectors. Trust was built through staff exchanges between the agencies of the different countries; the time spent at another’s agency allowed the visiting staff to learn how their counterparts undertook scientific reviews and regulatory activities. Also, dossiers were reviewed by assessors from different countries—through this work sharing and joint report preparation, the staff got to learn from each other and see one another’s expertise; this was also true when staff undertook joint inspections of manufacturing facilities. Finally, because joint assessments and inspections are generally considered more transparent and stringent than national assessments and inspections, because of the greater amount of expertise involved, Partner States tended to be confident in the resulting findings. At the same time, some manufacturers expressed concern that joint assessments imposed high international standards, whereas some national assessments did not [9]. For example, some NMRAs had historically waived the requirement to demonstrate bioequivalence for certain products undergoing national assessments, but this requirement was not waived for joint assessments, because demonstrating bioequivalence is an international best practice.

An original focus of the initiative was using the joint assessment process to help facilitate access to medicines for the conditions that represent the largest health burdens in the EAC, such as HIV, malaria, tuberculosis, lower respiratory infections, and diarrheal diseases. It was thought that the benefits of undergoing joint assessment could be used to entice manufacturers and importers to focus on these types of medicines. However, most of the products to treat these conditions are—or will be—eligible for WHO’s Prequalification Programme. Thus, it became evident that local registration through the WHO Prequalification–NMRA Collaborative Registration Procedure (in which all EAC Partner States participate) was the most efficient process for bringing these medicines to market. The EAC MRH Steering Committee then decided to invite manufacturers of certain types of medicines that are not eligible for the WHO Prequalification Programme, such as anticancer and antihypertensive medicines, to use the joint assessment process. This attracted a high proportion of these types of applications: from 2015, when joint assessments began, through 2017, 16% of 49 joint product applications were for oncology products, and 24% were for cardiovascular disease products.

Under the guidance of a recently convened working group led by Kenya’s NMRA, the initiative also drafted harmonized guidelines for pharmacovigilance, as well as a pharmacovigilance roadmap and business plan to guide activities in this area. In March 2019, the EAC Sectoral Council of Ministers approved the EAC Harmonized Compendium of Guidelines for Pharmacovigilance [11], and the initiative has also announced plans to expand into laboratory testing of medicines and postmarketing surveillance in the coming years, described in more detail in an accompanying paper in this collection, *Optimizing the East African Community’s Medicines Regulatory Harmonization initiative in 2020–2022*: *A Roadmap for the Future* [12].

One of the initiative’s most striking successes was its contribution to increased regulatory capacity in NMRAs across the region. When the initiative began, in 2012, Kenya, Tanzania, and Uganda had established semiautonomous NMRAs, but Burundi, Rwanda, and Zanzibar only had departments or boards housed within their ministries of health to regulate medicines. Today, Rwanda and Zanzibar have full-fledged semiautonomous NMRAs, as does the EAC’s newest Partner State, South Sudan, and Burundi is expected to gain approval for its own NMRA in the near future. Joint activities and the initiative’s twinning program (which paired Zanzibar’s NMRA with Kenya’s, Burundi’s with Tanzania’s, and Rwanda’s with Uganda’s) have helped the newer NMRAs benefit from the knowledge and expertise of the more mature NMRAs, while strengthening the relationships between Partner States. In addition, the initiative has overseen trainings dedicated to building regulatory capacity in various functions. These trainings, carried out by WHO and the Swiss Agency for Therapeutic Products (Swissmedic), have focused on topics such as medicines evaluation and registration, GMP, QMS, IMS, and pharmacovigilance.

### MRH: Works in progress

Although beginning to conduct joint activities was a major achievement, stakeholders have noted several challenges with the first joint assessments performed, including

- A lack of transparency regarding timelines, making it difficult for applicants to track their applications’ progress;
- Inadequate NMRA follow-up to applicants’ questions;
- Inadequate applicant follow-up to NMRAs’ questions;
- Failure of assessors to screen for errors or omissions early in the process, leading to avoidable queries later in the process;
- Poor communication with technical partners about the scheduling of joint assessments, making it challenging for the partners to provide support; and
- Delays in obtaining national registrations once a joint recommendation had been made.

Similar problems were noted for joint GMP inspections in the BCG evaluation in 2017. How the initiative addressed these and other problems is described in an accompanying paper in this collection [12].

Another goal the initiative continues to work toward is implementing a shared IMS that links all Partner States, as well as the EAC Secretariat. Without an IMS, it is difficult for NMRAs to track the progress of applications in their own countries, much less in neighboring countries. Thanks to the initiative, all EAC Partner States now have a functioning IMS, and there is general agreement that this has boosted efficiency and strengthened cross-department linkages. The newer NMRAs of Burundi, Rwanda, and Zanzibar, as well as Uganda’s NMRA, chose to create a novel platform with support from the initiative, whereas Kenya and Tanzania kept their legacy systems. Ultimately, the platforms are designed to allow as many processes as possible to be conducted online, through a single portal. However, to date, only Kenya’s system allows applications to be submitted electronically. Moreover, to link Partner States’ IMS platforms to one another, as well as to the EAC Secretariat, the initiative must develop systems that are interoperable. A Cooperation Framework was signed in May 2018 by the EAC’s Council of Health Ministers; in it, Partner States agreed to share information on marketing authorization and GMP certifications, demonstrating their commitment to data sharing [13]. How this sharing will take place efficiently in the absence of a linked IMS connecting Partner States remains a challenge.

One last goal still in progress is for each of the EAC’s NMRAs to implement a QMS, to ensure that its regulatory processes are conducted in a uniform and rigorous manner. One of the most meaningful ways that NMRAs attempted to confirm the quality of their work was by becoming International Organization for Standardization (ISO) certified (This is the measure of an agency’s QMS as judged against ISO standards. Meeting the requirements for ISO certification is a globally recognized accreditation of the maturity and functionality of the QMS of an organization, including medical products regulatory agencies). In 2014, the QMS Working Group, led by Kenya and assisted by WHO and Swissmedic, established a harmonized QMS technical document based on the ISO 9001:2008 standard. However, the new ISO 9001:2015 standard was released shortly after, and EAC countries are now adopting it instead. To date, Kenya, Tanzania, Zanzibar, and Uganda’s NMRAs are 9001:2015 ISO-certified, and Rwanda and Burundi are currently working toward 9001:2015 ISO certification. In addition, Tanzania’s NMRA is the first in Africa to attain designation by WHO as a maturity level 3 (out of a total possible score of 4) agency, denoting an agency with “a stable well-functioning and integrated system of oversight for medical products” [14]. Once QMS are in place across the region, Partner States will have greater confidence in the quality of their neighbors’ work, increasing trust and facilitating reliance on the documents and inspections of their regional NMRA colleagues.

### Continuing challenges for the EAC MRH initiative

**Fig 4:**
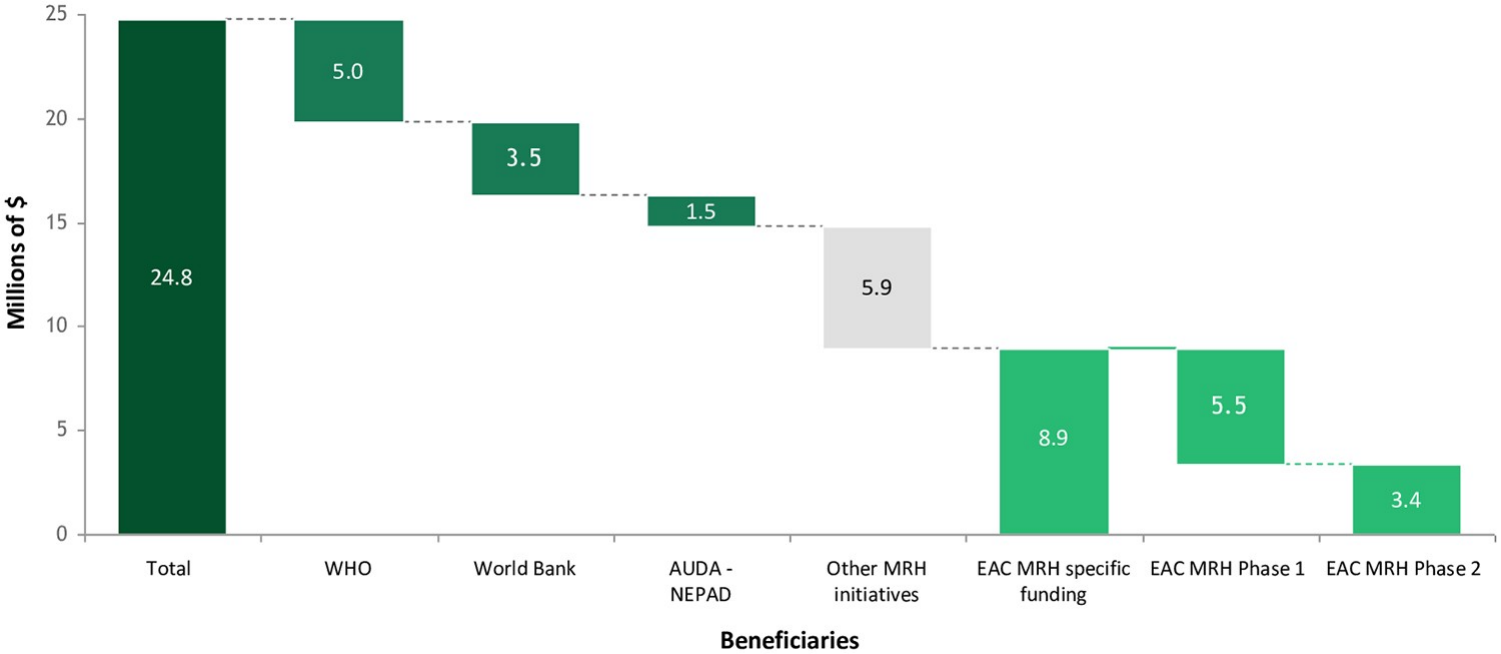
Breakdown of donor funding for the EAC MRH initiative, 2012–2017. EAC MRH Phase 1 lasted from July 2012 through July 2017, and Phase 2, which partially overlapped with Phase 1, lasted from July 2016 through December 2017. Light green represents EAC MRH–specific funding, medium green represents funding for partners, gray represents funding for other MRH initiatives (such as those in the Economic Community of West African States, South African Development Community, and Intergovernmental Authority on Development regions), and dark green represents total funding for the initiative. AMRH, African Medicines Regulatory Harmonization; AUDA-NEPAD, African Union Development Agency–New Partnership for Africa’s Development; EAC MRH, East African Community’s Medicines Regulatory Harmonization; WHO, World Health Organization.

A major goal of the EAC MRH initiative was to identify and implement a self-sustaining source of funding, so its activities could continue and even expand after initial catalytic donor funding ended, which was initially slated for 2017. Progress in this area has proved difficult. Originally, the plan was for Partner States to fund a growing portion of initiative activities starting several years after the program began. Little of such funding materialized, however. Ultimately, the Bill & Melinda Gates Foundation (BMGF), the UK Department for International Development, and the US Department of Treasury contributed almost US$25 million to the initiative between 2012 and 2017, with US$9 million directly supporting EAC activities and US$10 million supporting the activities of partners, such as WHO, World Bank, and AUDA-NEPAD, who were providing technical, financial, and advocacy assistance (Fig 4). Since then, the EAC Secretariat has received another $500,000 in support for the initiative from BMGF, as well as $750,000 from USAID to support pharmacovigilance work. At present, the well-established NMRAs of Kenya, Tanzania, and Uganda do support some program activities. For example, they fully cover the costs of their staff taking part in joint GMP inspections and also host initiative-related meetings. Heads of agencies have now also committed to covering most meeting costs, with donor funding covering only the costs of flying to face-to-face meetings going forward. The EAC MRH Steering Committee has indicated its commitment to transitioning to an EAC Medicines Agency- and fee-based funding system, but this process has not yet begun; future plans to address funding and other challenges are described in an accompanying article in this collection [12].

Another continuing challenge to conducting program activities has been bureaucratic inefficiencies. For example, the program’s donor trust fund is currently managed by the World Bank, which disburses funds to the EAC Secretariat, which then allots funds for program activities, many of which are conducted by NMRAs. Though both the World Bank and EAC Secretariat emphasize that their goal is to disburse funds as quickly as possible once they have received the required documentation, NMRAs report that complying with 2 sets of requirements is cumbersome and that, in some cases, delayed payments have led to joint activities being postponed or NMRAs having to advance money to conduct program activities. Another example of bureaucratic inefficiency is that, initially, the EAC Secretariat was responsible for hiring the officers stationed at NMRAs who were tasked with conducting key initiative activities. If one of these officers was not meeting their responsibilities at the NMRA, the Head of that NMRA had a difficult time holding him or her accountable, because the officer answered to the EAC Secretariat. This has been addressed by having project officers specifically focused on these joint activities appointed by and responsible to the individual heads of the EAC NMRAs.

Still another example of an inefficiency is that even after undergoing the joint assessment process, manufacturers must by law submit an application and fee to the NMRA of each EAC Partner State in which they want to register a medicine. The same goes for joint GMP inspections. The lack of a simple region-wide application process may discourage some medicines manufacturers and importers from swiftly registering their products throughout the EAC, especially in those Partner States that represent smaller markets.

One of the barriers to region-wide assessments and inspections is the lack of a binding legal framework that would require EAC Partner States to recognize the regulatory decisions of their neighbors. Originally, the initiative’s ultimate goal was for all NMRAs to formally commit to recognizing one another’s regulatory decisions. To date, however, the only such agreement is a unilateral one in which Zanzibar’s NMRA agrees to recognize the regulatory decisions of Tanzania’s NMRA. As mentioned, the EAC’s Council of Health Ministers signed a Cooperation Framework in May 2018, and this framework includes an agreement by Partner States to make regulatory decisions based on the outcomes of joint activities. This new agreement is nonbinding, however, and whether and how it will facilitate mutual recognition remains to be seen.

Given the importance of trust in Partner States collaborating with their regional NMRA partners and relying on each other’s work, the different capacities of the EAC’s NMRAs remain a challenge. The NMRAs of Burundi, Rwanda, and Zanzibar have made great strides in 5 short years, as has the NMRA of South Sudan, which joined the EAC in 2016. Burundi, for example, has received 630 applications since it began registering medicines in 2016, resulting in the registration of 216 medicines. Even so, the EAC’s more mature NMRAs sometimes need more assurance in the quality of their newer peers’ assessments and inspections. The lack of trust between NMRAs is not solely driven by experience level, however, as more mature NMRAs sometimes refuse to rely fully on each other’s decisions. To address some of these problems, Partner States have agreed to recognize a manufacturing site’s certifications if they come from 2 of the 3 most mature NMRAs in the EAC (those of Kenya, Tanzania, and Uganda). In general, however, even the less mature NMRAs would often rather improve their own capacity than rely on the work of more mature NMRAs, limiting opportunities for regulatory specialization, efficiency, and cooperation.

Staff turnover and understaffing also remain a challenge for the initiative. Since 2012, staff turnover in key leadership roles has been high. The enthusiasm and experience crucial to the initiative’s success wax and wane with changes in staff, posing a problem for the program’s continuity. Turnover also represents an important problem at the technical staff level for NMRAs, especially those with fewer resources. Burundi’s NMRA, for example, has trained 4 pharmacists to participate in product assessments since the initiative began, but all have left the agency for other missions, including positions in other ministerial departments or at international nongovernmental organizations. Having to constantly train new hires when trained staff leave, either because they have been assigned to other duties or because the private sector is eager to hire these individuals at higher salaries, is a major drain on the resources of the NMRAs and makes it difficult to conduct daily tasks smoothly. Activities associated with the EAC MRH initiative, which are often still perceived by NMRA staff as “extra” duties, are especially vulnerable to disruption.

Finally, many participants in the initiative believe that NMRAs, and especially the Heads of NMRAs, have yet to fully take up the mantle of leadership. To date, the EAC Secretariat, and sometimes program partners, have tended to shape the initiative’s agenda. Heads of NMRAs have expressed a desire for greater involvement in defining program priorities, as they are most familiar with the needs of their organizations and the health situations in their countries. Furthermore, leadership at the national level is critical to ensure that regulatory harmonization and optimization activities take place efficiently and become part of the everyday workflow at NMRAs. In particular, enthusiastic support by the Heads of NMRAs is needed to convince staff that the short-, medium-, and long-term benefits of regional cooperation outweigh the challenges. To nurture this type of leadership, the Heads of NMRAs have recently started convening their own meetings, in which they can candidly discuss future goals with one another and formulate work plans. This is a key step in ensuring that the initiative continues to grow and improve.

## Conclusion

In its pilot phase, the EAC’s MRH initiative reduced the amount of time it took to register medicines in individual countries by about half. It did so by instituting a suite of regulatory standards and processes aligned across the region, as well as by building the capacity of all its Partner States’ NMRAs to engage in regulatory activities. Thanks to the initiative, some medicines are also now being assessed jointly by the NMRAs of all EAC Partner States, helping to set the stage for a future in which a company can file a single application to market a product throughout the entire EAC. Joint GMP inspections are now being conducted as well, further contributing to regulatory efficiency in the region. There is more trust among and between experts in the region. However, the initiative still has much work to do, including shifting from relying on donor support to becoming self-sustaining, optimizing cooperation between Partner States with very different levels of resources and experience, and moving toward a more transparent system that is easier for applicants to navigate. In an accompanying article in this collection, *Optimizing the East African Community’s Medicines Regulatory Harmonization initiative in 2020–2022*: *A Roadmap for the Future* [12], we will describe the EAC’s plans for the next 3 years, as it seeks to learn from the lessons of the pilot phase and transition from a pilot project to an efficient, transparent, accountable, and permanent feature of the EAC regulatory landscape, including an expansion to pharmacovigilance.

## Abbreviations

AUDA-NEPAD: African Union Development Agency–New Partnership for Africa’s Development
BCG: Boston Consulting Group
BMGF: Bill & Melinda Gates Foundation
CTD: Common Technical Document
EAC: East African Community
GMP: good manufacturing practice
ICH: International Council for Harmonisation of Technical Requirements for Pharmaceuticals for Human Use
IMS: information management system
ISO: International Organization for Standardization
MRH: Medicines Regulatory Harmonization
NMRA: National medicines regulatory authorities
QMS: quality management system
SOP: standard operating procedure
Swissmedic: Swiss Agency for Therapeutic Products
WHO: World Health Organization
